# Research Review: Changes in the prevalence and symptom severity of child post‐traumatic stress disorder in the year following trauma – a meta‐analytic study

**DOI:** 10.1111/jcpp.12566

**Published:** 2016-05-12

**Authors:** Rachel M. Hiller, Richard Meiser‐Stedman, Pasco Fearon, Sarah Lobo, Anna McKinnon, Abigail Fraser, Sarah L. Halligan

**Affiliations:** ^1^ Department of PsychologyUniversity of BathBathUK; ^2^ Department of Clinical PsychologyUniversity of East AngliaNorwichUK; ^3^ Department of Clinical, Educational and Health PsychologyUniversity College LondonLondonUK; ^4^ Department of PsychologyMacquarie UniversitySydneyAustralia; ^5^ MRC Integrative Epidemiology UnitSchool of Social and Community MedicineUniversity of BristolBristolUK

**Keywords:** Child, adolescent, trauma, posttraumatic stress, meta‐analysis, longitudinal

## Abstract

**Background:**

Understanding the natural course of child and adolescent posttraumatic stress disorder (PTSD) has significant implications for the identification of, and intervention for, at‐risk youth. We used a meta‐analytic approach to examine longitudinal changes in youth PTSD prevalence and symptoms over the first 12 months posttrauma.

**Methods:**

We conducted a systematic review to identify longitudinal studies of PTSD in young people (5–18 years old), excluding treatment trials. The search yielded 27 peer‐reviewed studies and one unpublished dataset for analysis of pooled prevalence estimates, relative prevalence reduction and standardised mean symptom change. Key moderators were also explored, including age, proportion of boys in the sample, initial prevalence of PTSD and PTSD measurement type.

**Results:**

Analyses demonstrated moderate declines in PTSD prevalence and symptom severity over the first 3–6 months posttrauma. From 1 to 6 months posttrauma, the prevalence of PTSD reduced by approximately 50%. Symptoms also showed moderate decline, particularly across the first 3 months posttrauma. There was little evidence of further change in prevalence or symptom severity after 6 months, suggesting that it is unlikely a child would lose a PTSD diagnosis without intervention beyond this point**.**

**Conclusions:**

The current findings provide key information about the likelihood of posttrauma recovery in the absence of intervention and have important implications for our understanding of child and adolescent PTSD. Results are discussed with reference to the timing of PTSD screening and the potential role of early interventions. Findings particularly highlight the importance of future research to develop our understanding of what factors prevent the action of normal recovery from the ‘acute’ posttrauma period.

## Introduction

Trauma exposure in young people may result in the development of posttraumatic stress disorder (PTSD), a potentially highly persistent problem (e.g., Morgan, Scourfield, Williams, Jasper, & Lewis, 2003; Yule et al., 2000). PTSD is traditionally characterised by the presence of intrusive thoughts relating to the traumatic event, avoidance of reminders of the trauma and hyperarousal (e.g., difficulty in sleeping; American Psychiatric Association, 2000), while the most recent diagnostic manual also includes the presence of negative alterations in cognitions and mood (American Psychiatric Association, 2013). The development of PTSD in childhood or adolescence is associated with serious comorbid psychological difficulties, including depression, conduct problems and substance use, as well as poorer criminal justice outcomes (e.g., Steiner, Garcia, & Matthews, 1997). More broadly, it can have a significant impact on social, emotional and educational outcomes, thus presenting as a significant threat to a young person's developmental trajectory (e.g., Mathews, Dempsey, & Overstreet, 2009; McDermott, 2009).

While recent meta‐analyses have highlighted the cross‐sectional prevalence of child and adolescent PTSD in trauma exposed populations (11%–20%; Alisic et al., 2014; hereafter referred to as child PTSD), as well as risk factors for its development (Trickey, Siddaway, Meiser‐Stedman, Serpell, & Field, 2012), there are few empirically derived estimates as to the *course* of the disorder. This is surprising, given that both diagnostic algorithms (American Psychiatric Association, 2013; World Health Organization, 1992) and clinical guidelines (Cohen, 2009; NICE, 2005) assume a typical response to be an acute elevation of posttraumatic stress symptoms followed by some degree of natural recovery in the first months following trauma. However, while some longitudinal studies of child PTSD have reported the expected marked natural decreases in prevalence and symptom severity in the months following trauma (e.g., Pervanidou et al., 2007; Saxe et al., 2001), others have found little change in prevalence and symptom severity over time (e.g., Hitchcock, Nixon, & Weber, 2014; Landolt, Vollrath, Timm, Gnehm, & Sennhauser, 2005; Landolt, Ystrom, Sennhauser, Gnehm, & Vollrath, 2012). Understanding how PTSD prevalence and symptom severity may naturally change, in the absence of intervention, has significant implications for identification and treatment of child PTSD, including informing the timing and design of early screening and interventions, providing information about the likelihood of natural recovery and determining appropriate periods for ‘watchful waiting’.

We conducted a meta‐analytic study of the longitudinal literature to quantify PTSD prevalence over time and symptom severity change in children and adolescents, with a focus on the 1‐year period following a trauma. In particular, our primary aims were to examine change in prevalence and symptom levels in the year following trauma and determine whether there is a particular time point where PTSD prevalence and symptom severity plateau. To describe the sample, we also explored the point prevalence of PTSD at four posttrauma time points. As a secondary aim, we also conducted preliminary examinations of potential sample/methodological characteristics that may be associated with rates of change. We considered the following variables: the type of trauma, based on reports that PTSD rates are higher in the case of interpersonal trauma (e.g., assault; Alisic et al., 2014); the sample gender ratio, based on evidence that girls report higher PTSD symptoms than boys (e.g., Alisic et al., 2014; Martinez, Polo, & Zelic, 2014; Trickey et al., 2012); PTSD measurement via self‐report v interview, as these may differ in their reliability in detecting PTSD (e.g., Shalev, Freedman, Peri, Brandes, & Sahar, 1997); the mean age of the sample, with some, albeit inconsistent evidence, that prevalence may differ by age (e.g., Foy, Madvig, Pynoos, & Camilleri, 1996; Martinez et al., 2014); proportion of the sample with PTSD at the first time point, as a higher proportion potentially allows for steeper decline in symptoms; and whether PTSD in the ‘acute’ phase was measured within 4 weeks of the event or 4–6 weeks posttrauma. The latter was included as diagnostic guidelines preclude formal diagnosis of PTSD within 4 weeks of the event (American Psychiatric Association, 2013).

## Methods

### Sample of studies

The protocol for this review was preregistered on PROSPERO (CRD42014014544). The search was designed in close consultation with a University librarian. PsycARTICLES (which includes PsycNet and PsycInfo) and PubMed were searched for publications between 1980 (when PTSD was first introduced in the DSM) and September 2014. Search terms were ‘child’ (including all search engine variants) OR ‘adolescent’ (including all search engine variants), AND ‘posttraumatic stress’ OR ‘post traumatic stress’ OR ‘post‐traumatic stress’ AND ‘longitudinal’ OR ‘prospective’. Age filters were applied to search for samples of children aged 0–18 years. We also searched the reference lists of included articles for additional relevant studies and had the final list of articles reviewed by an expert in the child and adolescent PTSD field. Finally, research groups referenced twice or more in the final list of included studies were contacted to enquire about relevant unpublished work (i.e., recently completed longitudinal studies). From this process, only one unpublished dataset was received (from co‐author RMS; Meiser‐Stedman et al., unpublished).

Studies were included if they studied children aged between 5 and 18 years who had been exposed to a trauma^1^; they utilised a standard measure of PTSD (questionnaire and/or diagnostic interview); and they measured PTSD on at least two of the following time points: 1 month (approximately ±2 weeks; ‘acute PTSD’), 3 months (±1 month), 6 months (±1 month) and 1 year (±1 month) posttrauma. Studies that included only one measure of PTSD with an earlier measure of acute stress disorder were excluded from the analysis due to lack of symptom equivalence (Bryant, Creamer, O'Donnell, Silove, & McFarlane, 2008). The use of discrete study time points was essential to allow data collation and the exploration of symptom and prevalence change across discrete time periods. Time points were selected based on those covered in the vast majority of the longitudinal literature. If studies included additional time points (e.g., measured PTSD at 3 and 6 months and then again at 2 years posttrauma), data were only extracted on the specific time points of interest.

Articles were excluded if any of the following applied: the sample received an intervention, although nontreated trauma control groups could be included if otherwise appropriate; it was not clear what the elapsed time was between trauma exposure and measurement of PTSD^2^; and where articles reported only on a traumatic brain injury (TBI) sample, data were only extracted for children who had experienced a mild TBI, and not for those with moderate to severe TBI, due to difficulties differentiating more severe TBI symptoms from PTSD symptoms (Bryant, 2011).

**Figure 1:**
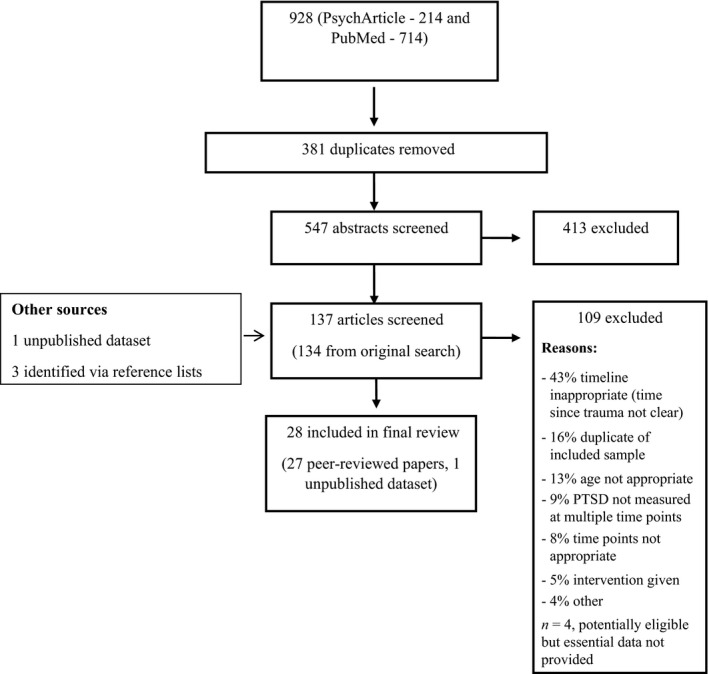
PRISMA diagram for study inclusion process

The identification of relevant articles followed PRISMA guidelines (Moher, Liberati, Tetzlaff, & Altman, 2009), as summarised in Figure 1. Co‐author SL and a trained research assistant reviewed the abstracts of all studies identified in the initial search, and RH then reviewed all excluded studies to ensure decisions were consistent with the inclusion/exclusion criteria. Agreement was found for 99.98% of excluded studies (i.e., all but one). The primary reasons for exclusion at this initial screen were that PTSD was measured only at a single time point or that the study addressed adult PTSD (e.g., adult survivors of child abuse). RH and co‐author AM then each independently reviewed the entire article for the remaining studies in relation to inclusion/exclusion criteria. This resulted in the exclusion of a further 109 articles, with excellent agreement (kappa = .79). For those articles where there was disagreement (*n* = 3), agreement was reached via a consensus meeting with co‐authors SH, RMS and PF. Thirty‐one peer‐reviewed articles initially met criteria for inclusion. For approximately 40% of articles, it was then necessary to contact corresponding authors to request relevant missing information. Subsequent to this, four of the eligible articles were ultimately not included as the authors were unable to provide essential data (Rivara, McCarty, Shandro, Wang, & Zatzick, 2014; Wang, Elhai, Dai, & Yao, 2012; Zonfrillo et al., 2014) or could not be contacted (Holbrook et al., 2005). Finally, co‐author (RMS) supplied an unpublished dataset from a recently completed large‐scale study (Meiser‐Stedman et al., unpublished).

In total, 27 peer‐reviewed published articles and one raw dataset were included in this review (see Table 1 for a complete list). Of this final set of 28 studies, all defined PTSD using the DSM‐IV/TR (American Psychiatric Association, 2000) with the exception of two (Di Gallo, Barton, & Parry‐Jones, 1997; La Greca, Silverman, Vernberg, & Prinstein, 1996), which used DSM‐III criteria (American Psychiatric Association, 1987).

**Table 1:** Overview of studies included in meta‐analysis

Author (year)	Country	Age, years range (mean)	% male	*N* a	Trauma type (most common)	Time point for analysis: time since trauma	Source	Instruments
Bryant, Mayou, Wiggs, Ehlers, and Stores (2004)	United Kingdom	5–16 (12.3)	55	81	Accidental injury (43% car accident)	2 weeks 3 months 6 months	Child	CIES; CPTSD‐RI
Coville and Pierce (2012)	United Kingdom	7–17 (11.2)	68	66	Admission to PICU	3 months 1 year	Child	CRIES
Di Gallo et al. (1997)	United Kingdom	5–18 (10.2)	67	50	MVA	<4 weeks 3 months	Child	CRIES; CPTSD‐RI
Doron‐LaMarca, Vogt, King, King, and Saxe (2010)	United States	6–18 (13.3)	75	157	Accidental injury (24% burns)	<4 weeks 3 month 1 year	Child	CPTSD‐RI
Hajek et al. (2010)	United States	8–15 (11.9)	63	285	TBI and orthopaedic injury (events NR)	<4 weeks 3 months 1 year	Parent	PCL‐C/PR
Hitchcock et al. (2014)	Australia	7–17 (11.9)	80	50	Accidental injury (46% falls)	1 month 3 months	Child	CPSS
La Greca et al. (1996)	United States	8–12 (NR)	42	442	Hurricane	3 months 7 months	Child	CPTSD‐RI
Landolt et al. (2005)	Switzerland	6.5–14.5 (9.8)	54	68	MVA	4–6 weeks 1 year	Child	CPTSD‐RI
Landolt et al. (2012)^b^	Switzerland	6.5–16 (10.4)	61	138	Accidental injury^b^	5–6 weeks 1 year	Child	CPTSD‐RI
Le Brocque et al. (2010)	Australia	6–16 (10.7)	63	169	Accidental injury	4–6 weeks 6 months	Child	CIES
Meiser‐Stedman et al. (2007)	United Kingdom	10–16 (13.8)	64	93	Assaults and MVA (assault, 57%)	<4 weeks 6 months	Child	ADIS‐PTSD
Meiser‐Stedman, Smith, Glucksman, Yule, and Dalgleish (2008)^c^	United Kingdom	7–10 (9.3)	54	52	MVA	<4 weeks 6 months	Child	CAPS‐CA
Meiser‐Stedman et al. (unpublished)	United Kingdom	8–17 (14.1)	58	226	Accidental injury (MVA, 46%)	<4 weeks 2 months	Child	CPTSDI
Mirza, Bhadrinath, Goodyer, and Gilmour (1998)	United Kingdom	8–16 (13.6)	64	119	MVA	4–6 weeks 6 months	Child	FRI
Nixon, Ellis, Nehmy, and Ball (2010)	Australia	7–17 (12.2)	61	90	Accidental injury (54% MVA)	3 months 6 months	Child	CAPS‐CA; CPSS
Nugent, Ostrowski, Christopher, and Delahanty (2007)	United States	8–18 (13.2)	61	82	Accidental injury and assault (50% MVA)	6 weeks 6 months	Child	CAPS‐CA
O'Connor et al. (2012)^d^	United States	14–17 (NR)	71	120	TBI (event NR)	3 months 1 year	Child	UCLA‐PTSD‐RI
Ostrowski, Christopher, van Dulmen, and Delahanty (2007)	United States	8–18 (13.4)	56	45	Accidental injury (56% MVA)	6 weeks 7 months	Child	CAPS‐CA
Pervanidou et al. (2007)	Greece	7–18 (11.0)	71	56	MVA	1 months 6 months	Child	K‐SADS‐PTSD; CPTSD‐RI
Saxe et al. (2001)	United States	6–16 (11.7)	54	24	Burns	<4 weeks 6 months	Child	CPTSD‐RI
Schafer, Barkmann, Riedesser, and Schulte‐Markwort (2006)	Germany	8–18 (13.6)	58	72	MVA	<1 week 3 months	Child	CIES
Self‐Brown, Lai, Thompson, McGill, and Kelley (2013)	United States	8–16 (11.6)	49	426	Hurricane	3 months 13 months	Child	UCLA‐PTSD‐RI
Sturms et al. (2005)	The Netherlands	8–15 (12.2)	53	49	Accidental injury (47% MVA)	3 months 6 months	Child	CIES (Dutch version)
Zatzick et al. (2006)	United States	12–18 (15.9)	68	108	Accidental injury and assault (89% accidental injury)	<4 weeks 2 months 5 months 1 year	Child	UCLA‐PTSD‐RI
Zehnder, Meuli, and Landolt (2010)^e^	Switzerland	7–16 (11.3)	58	50	MVA	<4 weeks 2 months 6 months	Child	CAPS‐CA (German version)
Zehnder, Prchal, Vollrath, and Landolt (2006)^f^	Switzerland	6–15 (9.8)	58	101	Accidental injury	5–6 weeks 1 year	Child	CPTSD‐RI (German version)
Zhang et al. (2012)	China	NR (16.9)	43	548	Earthquake	6 months 1 year	Child	PCL‐C
Zink and McCain (2003)	United States	7–15 (10.8)	60	143	MVA	2 months 6 months	Child	DICA‐R‐PTSD

NR, not reported; MVA, motor vehicle accident (this may include accidents where the child was a pedestrian or a passenger); TBI, traumatic brain injury; CIES, Impact of Event Scale – child version; CPTSD‐RI, Child PTSD Reaction Index; CRIES, Child Revised Impact of Event Scale; PCL‐C/PR, PTSD Checklist for Children/Parent Report; CPSS, Child Post‐Traumatic Stress Scale; ADIS‐PTSD, Anxiety Disorder Interview Schedule – PTSD Module; CAPS‐CA, Clinician Administered PTSD Scale for Children/Adolescents; FRI, Frederick's Reaction Index; UCLA‐PTSD‐RI, University of California Los Angeles PTSD Reaction Index; K‐SADS‐PTSD, Kiddie Schedule for Affective Disorders and Schizophrenia – PTSD; DICA‐R‐PTSD, The PTSD Diagnostic Interview for Children and Adolescents.

a *N* represents the total sample size at the first point where PTSD was formally assessed and for the relevant sample.

b Accidental injury sample only (Landolt et al., 2012).

c Older sample only (7‐ to 10‐year olds; Meiser‐Stedman et al., 2008).

d Only includes those in mild TBI group (O'Connor et al., 2012).

e Accidental injury sample only (Zehnder et al., 2006).

f Control group only (Zehnder et al., 2006).

### Data extraction

Data were extracted by RH and independently verified by SL. Information was collected on the year of publication, country of origin and key study characteristics (sample size, average age, age range, percentage of girls and boys, primary trauma type and PTSD assessment tool). Summary information for each study is presented in Table 1. Key outcome data extracted for each available time point were (a) the prevalence (number and proportion) of children who met PTSD criteria based on either a diagnostic interview or a cut‐off on a self‐report questionnaire; and (b) mean scores and standard deviations on questionnaire measures of PTSD symptom severity. Where both self‐report questionnaires and diagnostic interviews were used (Table 1), the prevalence of PTSD based on diagnostic interview was used. To explore whether the use of self‐report questionnaire versus diagnostic interview may have impacted the results for prevalence analyses, this was included as a potential moderator. Where data were collected based on both child self‐report and parent report, the young person's report was used, based on evidence that parents tend to under‐report child PTSD symptoms (Kassam‐Adams, Garcia‐Espana, Miller, & Winston, 2006; Meiser‐Stedman, Smith, Glucksman, Yule, & Dalgleish, 2007)^3^.

### Analyses

Meta‐analyses were conducted using STATA version 13 (StataCorp, 2013), using random effects modelling with 95% confidence limits for all estimates (Riley, Higgins, & Deeks, 2011). The ‘metan’ command (Harris et al., 2010) was used to establish the pooled prevalence of PTSD at each time point, the reduction in the number of children meeting criteria for PTSD between time points (referred to as ‘prevalence reduction’), and the strength of mean symptom change between time points (based on the pooled effect size). Based on the available data (see Table 1 for available time points for each study), we examined changes in pooled prevalence and mean symptom severity across four key posttrauma time frames: 1 to 3 months, 3 to 6 months, 1 to 6 months and 3 months to 1 year.

For analyses of point prevalence and prevalence change, we used logit transformations for better estimations but later transformed data back to proportions for ease of interpretation (Lipsey & Wilson, 2001). Absolute prevalence estimates are potentially misleading in relation to change over time, as different sets of studies may be represented at different time points. If there is significant heterogeneity in baseline levels of PTSD across different studies, variation in which studies can be included in the analyses for each time point means that a difference in absolute prevalence between two time points may not reflect actual change in PTSD prevalence. This problem does not arise in relation to prevalence or mean symptom change statistics, as estimated change is computed taking account of starting levels of PTSD within the study. To explore the change in the proportion of children with PTSD (‘prevalence reduction’), we computed the following for each study time point: 100% – (*n* with PTSD at T2/*n* with PTSD at T1), where T1 and T2 are the earlier and later time points, respectively. Thus, this represents the proportion of the sample who ‘lost’ their PTSD diagnosis between time points. In rare cases where there was no change in the proportion of children with PTSD, 0.01 (i.e., 1%) was added to allow for logit transformation. As this analysis only examined the subset of the sample who had PTSD at T1, standard errors and confidence intervals were calculated using the number of children with PTSD at that time point, rather than the whole sample^4^. Where there was attrition between time points, we assumed that the proportion of PTSD in those lost to follow‐up was the same as the proportion of PTSD at the initial time point. To examine the effect of this assumption, we also performed sensitivity analyses, which assumed either all drop‐outs had PTSD or no drop‐outs had PTSD.

For analyses of change in mean PTSD symptom scores, effect sizes were established using the change in the mean scores on the study's PTSD measure (Table 1) and the pooled standard deviation. These analyses could make use of the entire sample for each study. Further sensitivity analyses were performed to determine whether the pattern of results for symptom change would differ for varying correlations between baseline and follow‐up symptom severity, assuming correlations of 0.2, 0.4, 0.6 and 0.8 for each time lapse (i.e., 1–3 months, 1–6 months, 3–6 months and 3–12 months).

For all analyses, heterogeneity was quantified using estimates of I^2^, which captures the percentage of the total observed variability that is due to true prevalence differences between studies rather than chance variation (Higgins, Thompson, Deeks, & Altman, 2003). Where significant heterogeneity was identified between studies, meta‐regression was used to investigate the extent to which heterogeneity could be explained by the following prespecified characteristics (Harbord & Higgins, 2009): PTSD prevalence at the study's first time point (for change analyses only); measurement of PTSD (coded as [0] self‐report with cut‐off or [1] diagnostic interview; relevant for relative prevalence change and point prevalence analyses only); gender (coded as proportion of boys); average age of sample; and timing of the 1‐month PTSD measurement, coded as (0) if at <4 weeks posttrauma (i.e., in the acute stress period when PTSD is not formally diagnosed) or (1) at 4–6 weeks (i.e., when a diagnosis of PTSD is appropriate). The latter analysis was included to examine whether any change (or lack thereof) in prevalence or symptoms from the acute phase (1 month) was impacted by the inclusion of studies that measured PTSD at <4 weeks. The informant for PTSD symptoms and type of trauma were also considered a priori as potential determinants of PTSD prevalence and symptoms. However, these were ultimately not included, as only one study measured PTSD via parent report only, and there was insufficient power to compare trauma types, with the large majority of included studies reporting ‘accidental injury’ (e.g., motor vehicle accident; MVA) as the primary or sole trauma type. Similarly, moderator analyses were initially planned for socioeconomic status (SES), injury severity and ethnicity, but these sample characteristics were found to be reported infrequently and inconsistently across studies, precluding investigation. Overall sample sizes were generally small for completing moderator analyses. Therefore, these must be regarded as preliminary. We also explored possible publication bias for each analysis (i.e., preference for publications where PTSD rates were higher) by inspecting funnel plots. There was evidence of asymmetry for studies included in each point prevalence analysis and in the analysis of prevalence change between 1 and 3 months posttrauma. However, this stemmed from missing studies with high prevalence and small sample size and thus is unlikely to reflect a publication bias. There was no evidence of significant asymmetry for studies included in any other analysis.

## Results

### Data summary

Table 1 provides summary information for included studies. Studies included in each meta‐analysis are listed in the corresponding figures. Children were aged 5–18 years and sample sizes ranged from 24 to 548 children. The majority of studies recruited children after accidental injury (e.g., MVA and burns), while three studies included a proportion of children who had experienced an assault, and three studies were natural disaster samples (Table 1). Number of studies (*k*) per analysis ranged from *k* = 18 to *k* = 11 for prevalence estimates, *k* = 9 to *k* = 6 for prevalence change and *k* = 7 to *k* = 4 for mean symptom change scores, with 1‐year and 3‐month time points being less well represented throughout.

### Proportion of PTSD at each time point

**Figure 2:**
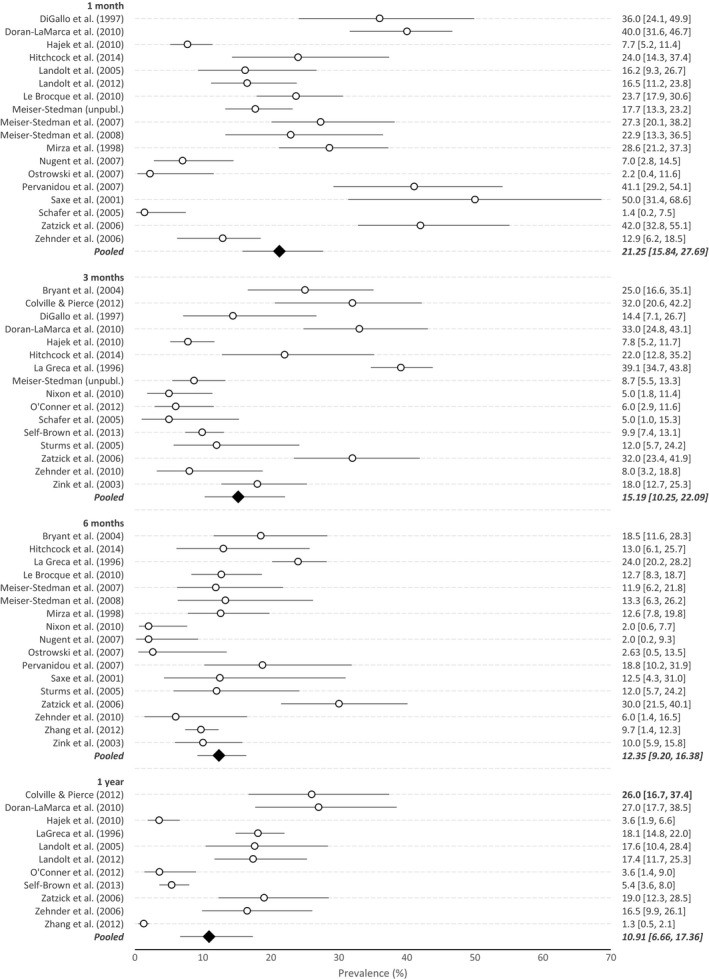
Plots of point prevalence (with 95% CI) of posttraumatic stress disorder at each of the four time points

Prior to the primary analysis of change, we explored the pooled point prevalence at each time point. In the acute stage (1 month; *k* = 18), the overall pooled PTSD prevalence was 21% (95% CI 16%–28%). The pooled prevalence at 3 months posttrauma (*k* = 16) was 15% (95% CI 10%–22%), while at 6 months posttrauma (*k* = 17), it was 12% (95% CI 9%–16%) and then 11% (95% CI 7%–17%) at 1 year posttrauma (*k* = 11). Forest plots for meta‐analyses of the point prevalence at each of the four time points are presented in Figure 2. Significant heterogeneity was present at all time points (all *I* ^2^ > 85%).

For the analyses of potential moderators, meta‐regressions using the logit transformed data indicated that none of the prespecified variables (i.e., proportion of boys, PTSD measurement via self‐report versus diagnostic interview, mean age, and, for the 1‐month assessment, timing <4 weeks vs. 4–6 weeks posttrauma) were significantly associated with prevalence at any time point (*p* = .10–.97).

### Prevalence reduction between time points

**Figure 3:**
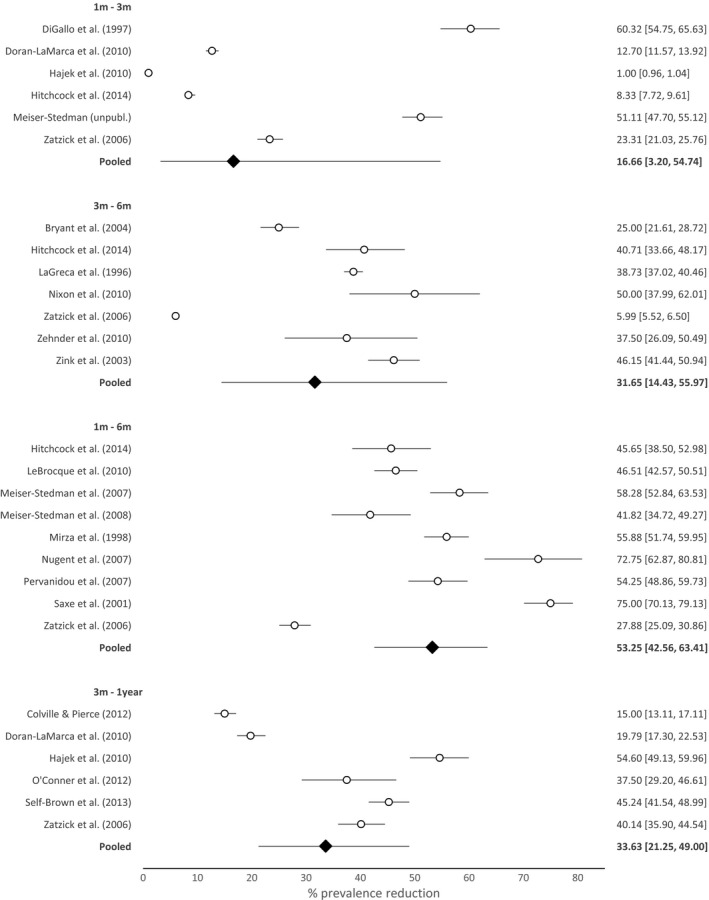
Plots of percentage reduction in posttraumatic stress disorder prevalence between time points (% change, 95% CI)

The results of meta‐analyses examining relative reduction in the proportion of children with PTSD across different time points are summarised in Figure 3. Analyses showed pooled prevalence reductions of 17% (95% CI 3%–55%) between 1 and 3 months (*k* = 6) and 32% (95% CI 14%–56%) between 3 and 6 months (*k* = 7). Over the longer time lapses, PTSD prevalence reduced by 53% (95% CI 43%–63%) between 1 and 6 months (*k* = 9) and 34% (95% CI 21%–49%) between 3 months and 1 year (*k* = 6). Heterogeneity was substantial in all analyses (all *I* ^2^ > 95%)^5^.

Analyses of potential moderators, using meta‐regressions and the logit‐transformed data, indicated that the proportion of boys in the sample, measurement type, initial PTSD prevalence and whether or not PTSD was first measured within 4 weeks of the trauma (for analyses including the 1‐month time point only) were not significantly associated with prevalence change (*p*s = .05–.94). Mean age was significantly associated with prevalence change between 3 and 6 months (*k* = 6; *p* = .01), with an older sample being associated with lower prevalence reduction (i.e., more stable prevalence).

### Effect sizes for changes in mean scores across time points

**Figure 4:**
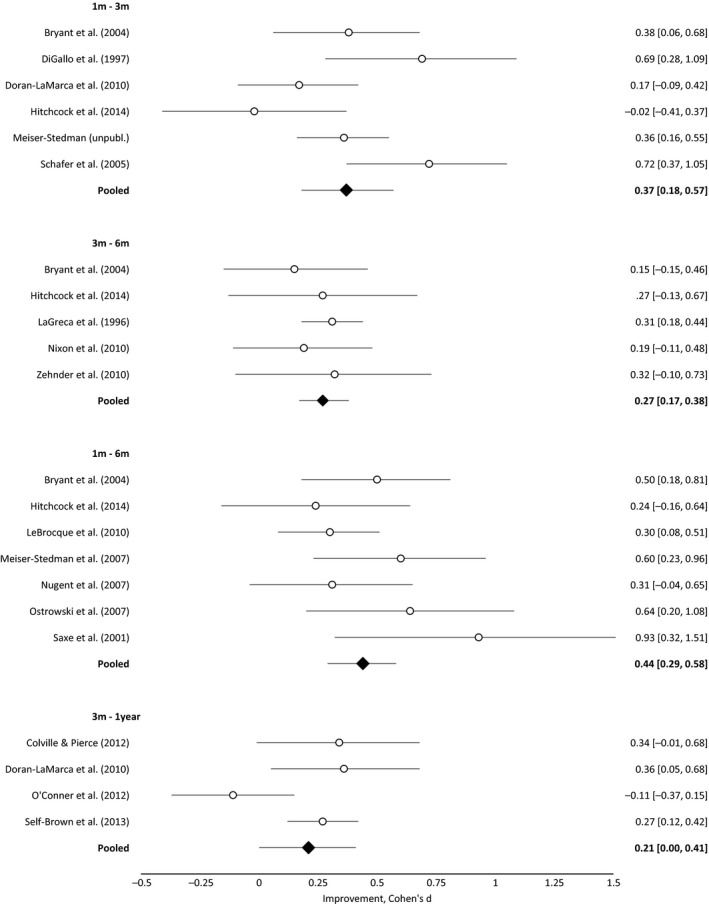
Strength of change in mean posttraumatic stress disorder symptom levels between time points (effect size, 95% CI)

Forest plots for the strength of the change in symptom severity are presented in Figure 4. Mean PTSD symptom scores significantly reduced across all time points (based on effect size confidence intervals). From 1 to 3 months (*k* = 6) and 1 to 6 months (*k* = 7), these reductions were small to moderate (*d* = 0.37 [95% CI 0.18, 0.57] and 0.44 [0.29, 0.58], respectively). From 3 months to the later time points (6 months [*k* = 5] and 1 year [*k* = 4]), there were small pooled reductions in symptom severity (*d* = 0.27 [0.17, 0.38] and 0.21 [0.00, 0.41], respectively). This pattern of results was robust to varying correlations between baseline and follow‐up symptom scores, confirmed by sensitivity analyses. There was statistically significant heterogeneity between 1 and 3 months (*I* ^2^ = 60.5%) but not for other intervals (1–6 months: *I* ^2^ = 18.1%; 3–6 months: *I* ^2^ = 0.0%; 3 months to 1 year: *I* ^2^ = 61.7%).

As there was evidence of significant heterogeneity for the 1‐ to 3‐month interval, the association with potential study variables was explored. However, we found no evidence that any variable was significantly associated with mean symptom change (i.e., proportion of boys, mean age, timing of the first PTSD assessment, initial proportion of PTSD; *ps* = .19–.94).

## Discussion

We used a meta‐analytic approach to estimate absolute prevalence and change in prevalence and symptoms of child PTSD in the year following trauma. Across all analyses, we found evidence of significant spontaneous reductions in both the prevalence and symptom severity of the disorder. PTSD prevalence showed significant decline across the first 6 months posttrauma, while symptom severity showed more marked improvement within the first 3 months and then only small declines thereafter. We largely failed to identify any consistent moderators of these changes, but given the limited power available these warrant further investigation in future work. The majority of studies were based on accidental injury samples, and the findings must necessarily be interpreted with this context in mind.

A recent meta‐analysis of cross‐sectional studies of child PTSD prevalence suggested an average prevalence of approximately 16% when collapsing across time points (Alisic et al., 2014). Our findings from the longitudinal literature build on this, showing that the headline figure of 16% likely masks marked variation over time. PTSD prevalence in the acute phase (1 month) posttrauma was 21%, declining to 15% at 3 months posttrauma and then to 12% and 11% at 6 months and 1 year posttrauma, respectively. This pattern is consistent with the assumption that a degree of spontaneous recovery is to be expected in children following trauma and suggests that this recovery proceeds at a slower rate after the first 3–6 months postevent.

Analysis of prevalence change across the first year posttrauma was broadly consistent with conclusions drawn from absolute prevalence estimates, i.e., indicating significant recovery up to 6 months. There was marked recovery for initial cases (53%) between 1 and 6 months posttrauma; recovery between 3 months and 1 year was also substantial (34%). Degrees of recovery for the shorter intervals (1–3 and 3–6 months) were correspondingly smaller, but broader confidence intervals mean that these estimates should be treated cautiously. Conclusions are somewhat limited by the fact that there was insufficient evidence available to examine prevalence change between 6 months and 1 year posttrauma (*k* = 2). Nonetheless, the findings overall are consistent in supporting a pattern of moderate recovery in the first few months posttrauma, after which problems are more likely to be stable.

Analysis of the course of PTSD symptom severity over the year posttrauma also demonstrated significant improvement over time, with effect size estimates across all time intervals indicating a degree of symptom reduction. Symptom change estimates showed a medium effect in terms of symptom reduction over the first 1–3 months posttrauma, a small‐medium effect size for the 1‐ to 6‐month interval, but only small effects for degree of symptom change between 3–6 months and 3 months to 1 year. All change estimates showed relatively wide confidence intervals (spanning small to medium effects), meaning that conclusions are necessarily tentative. However, the data support the conclusions from the prevalence analyses, suggesting significant symptom change over the first 3–6 months posttrauma, but modest change thereafter.

These findings have significant implications. Our analyses provide empirical support for the proposal that early traumatic stress reactions are relatively common in children and teens, and may recede – in more than 50% of initial cases – without formal intervention. For the period from 1 to 6 months posttrauma, this recovery was clear, regardless of whether PTSD prevalence or symptom severity was examined. Such findings are consistent with the few articles to explore child PTSD symptom trajectories, where some decline continues beyond the ‘acute’ phase (e.g., De Young, Kenardy, Cobham, & Kimble, 2012; La Greca et al., 2013; Le Brocque, Hendrikz, & Kenardy, 2010). Beyond 6 months posttrauma, there was then limited evidence for change in symptom severity or prevalence, although the smaller number of studies with a 1‐year follow‐up necessitates caution on this point. This widespread natural recovery observed in the first months posttrauma is an important feature of child and adolescent responses to trauma and warrants exploration.

Clinically, the current findings have multiple ramifications. The evidence for significant natural recovery occurring up to 6 months posttrauma suggests that careful thought is required when designing and implementing both screening and intervention programmes. First, in terms of screening, the diagnostic evidence presented here suggests that a screen for PTSD at 3–6 months posttrauma is substantially more likely to identify children who require intervention than an equivalent assessment completed within the first month posttrauma. The analysis of mean symptom levels is also consistent with this conclusion, but suggests more specifically that screening at 3 months may be effective, given that symptom decline begins to level off beyond this point. Nonetheless, although this might be the most cost‐effective way of detecting children needing treatment for PTSD, it may disadvantage individuals experiencing extremely high levels of symptoms in the acute phase by making them wait unnecessarily. Effective self‐screening, referral tools and screening thresholds are required to identify and distinguish disabling *acute* responses (that warrant immediate treatment) and potentially persistent PTSD symptoms that require further monitoring (i.e., ‘watchful waiting’).

In treatment terms, there is currently limited evidence to support the use of universal early interventions such as debriefing (Kramer & Landolt, 2011). Moreover, some commentators have argued that ‘over‐pathologising’ early reactions to trauma may reduce people's confidence in their own coping abilities (Wessely & Deahl, 2003). The current analyses highlight the need to consider whether any universal treatment delivered within the first 3 months posttrauma is likely to outperform the natural recovery process in children; consideration should also be given to the available resources and economic cost of such interventions (Scheeringa, Cobham, & McDermott, 2014). Early intervention approaches may particularly need to take account of the potential of children and adolescents to demonstrate resilience and should strive as far as possible to not deprive youth of the opportunity to develop their own mastery in the face of trauma. Nonetheless, low‐intensity approaches that facilitate existing support structures and can be delivered at low cost may be effective in helping families to understand normal and problematic child psychological responses to trauma and to identify children experiencing persistent psychological difficulties (e.g., Marsac, Donlon, & Berkowitz, 2014). Community‐ or service‐led early support programmes may also provide the foundation for targeted and/or stepped‐care interventions later on (Jaycox et al., 2010; Kramer & Landolt, 2011). It is also the case that early‐targeted intervention may be appropriate for child populations at particular risk of chronic PTSD (NICE, 2005), such as those who have been bereaved by trauma (Pfefferbaum et al., 1999), who have significant previous trauma histories (Copeland, Keeler, Angold, & Costello, 2007) or who evidence a strong profile of maladaptive cognitions and coping in the acute aftermath (Meiser‐Stedman, Dalgleish, Glucksman, Yule, & Smith, 2009; Stallard & Smith, 2007). This possible approach to targeting early intervention has not been systematically tested, and such at‐risk groups are not necessarily represented in the current analysis.

Given that traumatic stress symptoms are a relatively common response to trauma, the focus for intervention may be trying to understand what factors are preventing the action of normal recovery mechanisms. The current evidence particularly suggests that the aetiology of PTSD in children and teens may be better understood by differentiating the factors which underpin the onset of PTSD after a trauma and are involved in the substantial early recovery from PTSD versus the maintenance of difficulties. Thus, future research may be usefully directed not only at identifying the appropriateness of timing for the treatment of PTSD symptoms but also at discerning factors that differentiate children with acutely elevated PTSD symptoms who naturally recover from those who do not. For example, La Greca, Silverman, Lai, and Jaccard (2010) identified less social support, high anxiety and poor emotion regulation as key predictors of whether elevated PTSD symptoms would recede or remain chronic. Such studies may inform both the identification of at‐risk groups for chronic difficulties and the processes that are intervention targets.

Several features of the studies included in the current meta‐analysis warrant particular consideration. First, as already noted, the majority of studies focused on accidental injury and non‐intentional trauma exposures. As such, the available evidence base does not represent, arguably, those children who may be most vulnerable to persistent problems. Second, samples were derived from high‐income countries, predominantly European countries and the United States. Longitudinal studies of youth from low‐ and middle‐income countries are urgently needed, particularly given that ongoing stressors are more likely in such populations and access to psychological services is simultaneously restricted. Third, sociodemographic characteristics were not reported consistently across studies, and therefore, we were not able to include this in moderator analyses, but it is a potentially important determinants of outcome (e.g., La Greca et al., 1996; Trickey et al., 2012). Overall, it is essential that the evidence base in relation to child psychological responses to trauma is broadened to represent the wider population of trauma‐exposed youth.

## Limitations and research recommendations

This is the first article to use a meta‐analytic approach to estimate changes in prevalence and symptom severity of child PTSD over time. However, we acknowledge some limitations. First, there was a high amount of heterogeneity for most analyses, and we were largely unable to identify moderators to explain this. This is important, as it demonstrates the potential for different trauma populations to experience patterns of response and recovery that may deviate from those presented here. The heterogeneity between the studies may relate to differences between study samples (e.g., in terms of severity of events included) and methodologies; and the modest number of studies likely played a part in the failure to identify significant moderators. Other sample characteristics, including trauma type, SES and ethnicity, were considered a priori as potential moderators, but ultimately could not be explored. Moderator analyses must be considered preliminary, and identifying sources of heterogeneity should be considered a priority for future research. Measurement of PTSD was also variable, and we had to exclude some studies that measured ASD symptoms initially and PTSD symptoms later due to nonequivalence of measures. As such, another research recommendation from this article would be to assess PTSD symptoms and diagnosis (ignoring the requirement that symptoms should be present for at least 4 weeks) within the acute posttrauma period to allow accurate exploration of PTSD trajectories and thus assist in identifying those children who are less likely to naturally recover from acutely elevated symptoms. Further studies including follow‐up beyond 6 months are also needed.

A second potential limitation was that in examining prevalence and symptom severity changes over time, we could only focus on articles where PTSD was measured at a clearly specified time point relative to the trauma. This meant that studies including more chronic exposures, such as war trauma and child abuse, were excluded. Findings cannot be generalised to such populations. The large majority of studies were also accidental injury studies, and there were insufficient studies to compare the impact of accidental injury traumas versus interpersonal trauma or natural disasters. It is possible that varying posttrauma factors may mean symptom trajectories differ for different traumas. For example, following natural disaster or war trauma, the child and family may be faced with continued instability and safety concerns compared with children who experience a single‐incident motor vehicle collision. When more research becomes available on such populations, it would be important to explore whether symptom trajectories may differ.

Third, because this meta‐analysis explored change between time points, some necessary and carefully considered assumptions were required. To estimate change in prevalence, we had to assume that the children who met criteria for PTSD at later time points were a subset of those who met criteria at earlier time points. This assumption was consistent with the literature on PTSD symptom trajectories, where a delayed PTSD trajectory occurs in a very small percentage of children (e.g., 2%; De Young et al., 2012). We also had to assume that the prevalence of PTSD was equivalent in those children who dropped‐out between time points. To account for these necessary assumptions, we also performed sensitivity analyses and provided data on mean symptom changes (which included the mean for the whole sample) and point prevalence at each time point; these analyses yielded similar conclusions to the analysis of prevalence change. Nevertheless, it is the case that the extent of individual variation cannot be fully captured in a meta‐analytic study. While it might also be argued that the majority of papers' use of self‐report rather than diagnostic interview brings into question the reliability of findings, it is important to note that our preliminary analysis showed that the type of measurement failed to account for differences in either the point prevalence or the rate of prevalence change or symptom reduction.

## Conclusion

Overall, findings support clinical guidelines that there is significant natural recovery expected from the ‘acute’ posttrauma period to later ‘chronic’ periods. From 1 to 6 months posttrauma, the number of children with PTSD reduced by approximately 50%. In the absence of intervention, mean symptom levels changed to a small to moderate degree in the first 3 months posttrauma and plateaued from 3 months posttrauma. Results particularly highlight the need for research to focus on what factors may impact whether or not a child will fail to lose their ‘acute’ PTSD diagnosis status. Such research would allow for the appropriate use of resources to target those children where symptoms would be expected to remain chronic, while also providing insight in to why some children will naturally recover.

**Key points**

- Clinical and diagnostic guidelines suggest a natural decline in child posttraumatic stress disorder (PTSD) symptoms from the acute posttrauma stage is typical in the absence of intervention. However, there is little consensus in the literature about the appropriateness of such guidelines.
- Using a meta‐analytic approach, we found that PTSD prevalence reduces by approximately 50% over the first 6 months posttrauma. Symptom severity also showed moderate decline, particularly in the first 3 months posttrauma.
- There was little evidence of further change in prevalence or symptom severity after 6 months, suggesting that it is unlikely a child would lose a PTSD diagnosis without intervention beyond this point.
- The results have significant implications for the timing of screening and early intervention programmes for youth PTSD.

## Notes

1 This age range was chosen based on DSM‐IV‐TR and DSM‐5 guidance that PTSD may present differently for children under 6 years and given the limited empirical evidence relating to the presentation of PTSD in this group. A lower limit of 5 years was used as several studies included children as young as 5, yet mean age of the sample indicated that the majority of participants were older.

2 This criterion particularly resulted in the exclusion of articles on childhood sexual abuse (e.g., Feiring, Taska, & Chen, 2002), war trauma (e.g., Karam et al., 2014) and chronic illness, as it was not possible to pinpoint a specific exposure/stressor, and consequently, it was unclear how much time had lapsed since the trauma (vs. e.g., the end of conflict or disclosure of abuse) prior to the measurement of PTSD. Note, the DSM 5 also states that illness diagnosis should not necessarily be considered a criterion A trauma (American Psychiatric Association, 2013).

3 Only one study included only parent report on child PTSD symptoms (Hajek et al., 2010). As the inclusion of this study did not alter the pattern of results, it was retained in the analysis.

4 Note that one study was necessarily excluded from prevalence change estimates for 1–3 months and another for 1–6 months as each only had one participant initially with PTSD.

5 For sensitivity analysis, based on the assumption that *all* drop‐outs had PTSD, produced estimated pooled prevalence reductions were as follows: 1–3 months, 7.24% (2.37, 19.94); 3–6 months, 9.71% (2.48, 31.22); 1–6 months, 5.63% (2.90, 10.72); and 3 months to 1 year, 5.79% (2.29, 13.82). Note, in cases where this sensitivity analysis resulted in more PTSD at the earlier time point, the change was coded as no change in PTSD rates and then recoded as .01 (1% change) to allow for computation. Based on the assumption that *no* drop‐outs had PTSD, pooled prevalence reductions were as follows: 1–3 months, 28.09% (9.80, 58.42); 3–6 months, 35.43% (21.08, 53.25); 1–6 months, 59.12% (50.23, 67.48); 3 months to 1 year, 40.01% (26.70, 55.48).
